# Macrophages may promote cancer growth via a GM-CSF/HB-EGF paracrine loop that is enhanced by CXCL12

**DOI:** 10.1186/1476-4598-9-273

**Published:** 2010-10-14

**Authors:** Antonella Rigo, Michele Gottardi, Alberto Zamò, Pierluigi Mauri, Massimiliano Bonifacio, Mauro Krampera, Ernesto Damiani, Giovanni Pizzolo, Fabrizio Vinante

**Affiliations:** 1 Department of Medicine, Section of Hematology, University of Verona, Verona, Italy; 2 Department of Pathology and Diagnostics, Section of Pathological Anatomy, University of Verona, Verona, Italy; 3 Proteomics and Metabolomics Unit, Institute for Biomedical Technologies, CNR, Milan, Italy; 4 Department of Experimental Biomedical Sciences, University of Padua, Padua, Italy

## Abstract

**Background:**

Increased numbers of tumour-associated macrophages correlate with shortened survival in some cancers. The molecular bases of this correlation are not thoroughly understood. Events triggered by CXCL12 may play a part, as CXCL12 drives the migration of both CXCR4-positive cancer cells and macrophages and may promote a molecular crosstalk between them.

**Results:**

Samples of HER1-positive colon cancer metastases in liver, a tissue with high expression of CXCL12, were analysed by immunohistochemistry. In all of the patient biopsies, CD68-positive tumour-associated macrophages presented a mixed CXCL10 (M1)/CD163 (M2) pattern, expressed CXCR4, GM-CSF and HB-EGF, and some stained positive for CXCL12. Cancer cells stained positive for CXCR4, CXCL12, HER1, HER4 and GM-CSF. Regulatory interactions among these proteins were validated *via *experiments *in vitro *involving crosstalk between human mononuclear phagocytes and the cell lines DLD-1 (human colon adenocarcinoma) and HeLa (human cervical carcinoma), which express the above-mentioned ligand/receptor repertoire. CXCL12 induced mononuclear phagocytes to release HB-EGF, which activated HER1 and triggered anti-apoptotic and proliferative signals in cancer cells. The cancer cells then proliferated and released GM-CSF, which in turn activated mononuclear phagocytes and induced them to release more HB-EGF. Blockade of GM-CSF with neutralising antibodies or siRNA suppressed this loop.

**Conclusions:**

CXCL12-driven stimulation of cancer cells and macrophages may elicit and reinforce a GM-CSF/HB-EGF paracrine loop, whereby macrophages contribute to cancer survival and expansion. The involvement of mixed M1/M2 GM-CSF-stimulated macrophages in a tumour-promoting loop may challenge the paradigm of tumour-favouring macrophages as polarized M2 mononuclear phagocytes.

## Background

Over the last few years, a great deal of attention has been paid to the clinical significance of macrophages that infiltrate cancer. A number of studies provide evidence that tumour-associated macrophages are a negative prognostic factor of survival [1,2]. A recent gene-profiling study demonstrates that the overexpression of a macrophage signature and an increased number of tumour-infiltrating macrophages in diagnostic lymph-nodes are associated with poor outcome in classic Hodgkin's lymphoma patients [3]. Other studies underline pathways leading to M2 macrophage responses that foster tumour growth [4-7]. In the end, all these studies deal with the crosstalk between tumour cells and macrophages. For instance, a regulatory loop between breast cancer cells and macrophages has been described [8], and the cellular expression of matrix metallopeptidase 11 seems to be relevant to disease outcome at least in classic Hodgkin's lymphoma [3]. However, the grounds on which the above-mentioned prognostic significance rests are not so thoroughly appreciated, especially in terms of cell-to-cell molecular mechanisms.

Within the tangle of relations between macrophages and cancer cells, we tried to tease out the role that CXCL12 plays in both cancer cells and macrophages at the boundaries between cancer and inflammation. A tissue with high expression of CXCL12 (for example, liver or bone marrow) may represent a site that preferentially attracts both macrophages [9] and cancer cells [10,11], which co-migrate depending on their expression of the CXCL12 receptors CXCR4 and/or CXCR7 [12]. Ligand binding to these receptors, which are heterotrimeric guanine nucleotide-binding protein-coupled receptors (GPCR), activates matrix metallopeptidases that cleave EGF-family ligands, such as EGF or HB-EGF, from the cell membrane [13], leading to *trans*activation of HER1 on neighbouring cells [14]. This transactivation mechanism is a general function of GPCR signalling [15]. HER1 expressed by epithelial cancers plays a pivotal role by transducing signals that favour tumour progression [16,17]. The macrophage-regulator GM-CSF, which is produced by some types of cancer cells [18,19], specifically induces HB-EGF in macrophages and neutrophils [20].

Because mononuclear phagocytes express both CXCL12 GPCRs and HB-EGF, we argued that the recruitment of mononuclear phagocytes to a site of metastasis such as liver through CXCL12 should induce a release of HB-EGF, which is expected to activate HER1 and favour tumour progression. We found that tumour-associated macrophages and metastatic HER1-positive colon cancer in liver biopsies expressed a ligand/receptor repertoire that was consistent with our hypothesis and that *in vitro *CXCL12 could trigger a GM-CSF/HB-EGF paracrine loop whereby mononuclear phagocytes support cancer survival.

## Methods

### Ethical requirements

The blood and histological samples used in our study were in compliance with Institutional Review Board regulations.

### Cells and reagents

Highly purified human mononuclear phagocytes and neutrophils were isolated from the buffy coats [21] of blood samples from healthy volunteers. HeLa (human cervical carcinoma), DLD-1 (human colon adenocarcinoma) and Balb/c 3T3 (Swiss mouse embryo) cell lines (purchased from ATCC, Manassas, VA) and HUVEC (human umbilical vein endothelial cells, purchased from Cambrex, Walkersville, VA) were also used. Non-adherent and adherent cells were grown in RPMI-1640 medium and DMEM or TC199 + 10% FCS (complete medium; Invitrogen, Carlsbad, CA), respectively. Cells were treated with 200 ng/mL CXCL12 (Peprotech, London, UK) or 25 ng/mL GM-CSF (Genetics Institute, Boston, MA) or 25 ng/mL HB-EGF or 100 μg/mL anti-HB-EGF or 100 μg/mL anti-GM-CSF neutralising monoclonal antibody (mAb) (R&D Systems, Minneapolis, MN) or isotypic control immunoglobulins. After growing in cultures for the appropriate times in different conditions, the cells were either lysed for total RNA extraction or used for functional assays. In some experiments, the conditioned medium was replaced with fresh medium after 24 hours of stimulation and the cells were then maintained in culture for up to 48 hours. Cell-free supernatants (SN) were stored at -80°C.

### Immunochemistry on tissues and cells

Histological samples were obtained by hepatic lobectomy to excise metastatic nodules derived from colon cancer. After surgical excision, samples were put in buffered formalin, treated in an automated processor and embedded in paraffin. Four micrometre-thick slices were cut from paraffin blocks onto adhesive-coated slides. Cytological samples were obtained by allowing cells to grow on the slides. Antibodies (Ab) used included the following: CD163 (clone 10D6, 1/200; Novocastra, Newcastle-upon-Tyne, UK), CXCL10 and CXCR4 (both rabbit polyclonal, 1/500 and 1/100, respectively; Abcam, Cambridge, UK), CXCL12 (clone 7918, 1/100), GM-CSF (clone 3209, 1/100), HER1 (1/100), HER4 (1/100), and HB-EGF (clone 125923, 1/200) (all purchased from R&D Systems). Antigen retrieval was performed for all antibodies in a hot bath for 30 minutes at pH 6 (except for GM-CSF retrieval, which was performed at pH 8). For GM-CSF and HB-EGF no H_2_O_2 _blocking was performed. As controls, sequential sections or cytological slides were incubated with the Ab diluent and indifferent isotypic Ab. All procedures were performed on an automated stainer (Bond, Vision Biosystems, Melbourne, AU) using a polymer detection system (NovoLink, Novocastra).

### Flow cytometry

Following stimulation for 20 minutes or 24 hours with CXCL12, 1 × 10^6 ^cells were incubated with a goat anti-human HB-EGF IgG polyclonal Ab (Oncogene, San Diego, CA) or an IgG control Ab. After washing, 5 μL of phycoerythrin-conjugated swine anti-goat IgG (Caltag Laboratories, Burlingame, CA) was added. The cells were then analysed on a FACSCalibur (Becton-Dickinson, Mountain View, CA) using the FlowJo 8.8.2 program (Tree Star, Ashland, OR).

### HER1 transactivation assay

Cellular release of HB-EGF was evaluated by measuring its effect on HER1 phosphorylation at tyrosine 1068 (Y1068) using HeLa and DLD-1 as target cells. Approximately 3 × 10^6^effector cells (mononuclear phagocytes and neutrophils) were seeded in the upper chamber of a 0.4 μm transwell co-culture system (Costar, Cambridge, MA) and were stimulated with CXCL12 for 20 minutes. In a separate experiment, 3 × 10^6^ mononuclear phagocytes and neutrophils were cultured in the presence or absence of CXCL12 for 24 hours and their conditioned medium was added to the target cells for 20 minutes. HeLa cells were also stimulated for 20 minutes with recombinant human HB-EGF. Subconfluent HeLa cells that had been starved for 24 hours were incubated in the presence or absence of the anti-HB-EGF neutralising Ab (R&D Systems). Target cells were harvested for protein extraction.

### Protein extraction

Cell pellets were lysed for 30 minutes in 1 mL ice-cold cell extraction buffer (Biosource, Camarillo, CA), which was supplemented with a protease inhibitor cocktail (Sigma, St. Louis, MO) and 1 mM PMSF (Sigma). After centrifugation at 13,000 rpm for 10 minutes at 4°C, aliquots of the SN were stored at -80°C.

### Mass spectrometry

Approximately 1 × 10^7^ HeLa cells, which were either untreated or treated with HB-EGF for 20 minutes, were harvested for HER1 immunoprecipitation, reduction, alkylation and tryptic digestion. Ten microliters of the peptide mixture was analysed by means of an LCMS/MS system [22]. The data handling for the identification of phosphorylated residues was performed according to Guo et al. [23]. HB-EGF-induced HER1 phosphorylation was evaluated at Y992, Y1045, Y1068, Y1086, S1142, Y1148, Y1173, and expressed as phosphorylation ratio (phosphorylation after stimulus/basal phosphorylation).

### ELISA

*1. HER1 pY1068 and ERK1/2 pTpY185/187*. Total HER1 versus HER1 pY1068 and total ERK1/2 versus ERK1/2 pTpY-185/187 were measured in cell protein extracts using a commercially available ELISA (Biosource). The results were calculated as the ratio of phosphorylated molecules to total molecules and expressed as the percentage of phosphorylation. *2. HB-EGF and GM-CSF*. The cell-free culture SN was assayed for soluble HB-EGF protein using a specific ELISA developed in our laboratory [20] and for GM-CSF using a commercial ELISA (R&D Systems). Only SN previously purified from HB-EGF or GM-CSF by specific crosslinking (Invitrogen) were subsequently used as agonistic SN to induce HB-EGF or GM-CSF, respectively, in cell stimulation experiments followed by ELISA tests. *3. Apoptosis*. 72-hour internucleosomal DNA fragmentation as an index of apoptosis was evaluated using a cell death detection ELISA (Roche Diagnostics, Mannheim, DE) and expressed as the mean ± SD of triplicate determinations of the enrichment factor (absorbance of unstimulated cells/absorbance of stimulated cells).

### Northern blotting

Total RNA was extracted from the indicated cells. Northern blot analysis (with 10 μg RNA/lane) was performed as described [19]. Filters were hybridised with ^32^P-labelled cDNA probes complementary to the HB-EGF RNA sequence, which were generated in our laboratory or with a ^32^P-labelled plasmid containing a cDNA probe that detected the β-actin RNA sequence.

### RT-PCR

Total cellular RNA was purified by the QIAamp RNA Blood mini kit (Qiagen, Hilden, DE) + DNase digestion (Qiagen). Templates were generated from 2 μg of RNA with the Superscript III First-Strand Synthesis System kit (Invitrogen). cDNA for GM-CSF was amplified using the following primers (Invitrogen): 5'-CTCAGAAATGTTTGACCTCCAG-3' (sense) and 5'-TGACAAGCAGAAAGTCCTTCAG-3' (antisense) for a 221 bp amplicon. The GAPDH internal control primers (SuperArray Bioscience, Frederick, MD) were used to co-amplify a specific fragment that was 436 bp long. After 30 cycles at 95°C for 15", 55°C for 30" and 72°C for 30" in a Veriti thermal cycler (Applied Biosystems, Foster City, CA) using the AmpliTaq Gold PCR master mix (Applied Biosystems), the reactions were analysed by agarose gel electrophoresis and recorded using a VersaDoc imaging system (Bio-Rad, Hercules, CA).

### Cell proliferation and apoptosis

Hela, DLD-1, HUVEC and Balb/c 3T3 cells were seeded in 96-well plates in complete medium and allowed to adhere for 24 hours. They were then incubated in the absence or presence of the anti-HB-EGF neutralizing Ab (R&D Systems) and were stimulated with 25 ng/mL HB-EGF. In selected experiments, HeLa and DLD-1 were deprived of serum and cultured in the absence or presence of HB-EGF. Cell proliferation was measured by MTT incorporation [19] at 24, 48 and 72 hours and expressed as the proliferation index. Apoptosis was measurerd as the internucleosomal DNA fragmentation enrichment factor at 72 hours (see ELISA).

### siRNA knockdown

To silence the expression of GM-CSF, HeLa and DLD-1 cells were seeded on glass slides (10^5 ^cells/mL), stimulated with 200 ng/mL CXCL12 and/or 25 ng/mL HB-EGF or SN from GM-CSF-stimulated mononuclear phagocytes and transfected 48 hours using HiPerFect Transfection Reagent and siRNA (2 nM and 5 nM respectively, GM-CSF siRNA no. SI03037272 and AllStars negative control siRNA - Alexa Fluor 488; Qiagen). Transfection efficiency (> 95%) was determined by flow cytometry on trypsinized cells. Effective silencing of GM-CSF was determined by testing the induction and release of *1. GM-CSF in cancer cells *(using immunohistochemistry and ELISA, after a 24-hour stimulation with HB-EGF or mononuclear phagocytes conditioned medium) and *2. HB-EGF in mononuclear phagocytes *(using flow cytometry and ELISA, after a 24-hour stimulation with silenced cancer cell SN).

### Statistics

Student's *t*-test, Mann-Whitney U test and Kruskall-Wallis ANOVA by ranks were used. Differences were considered significant for *p *values < 0.05.

## Results

### Macrophages infiltrate colon cancer metastases in liver

**Figure 1:**
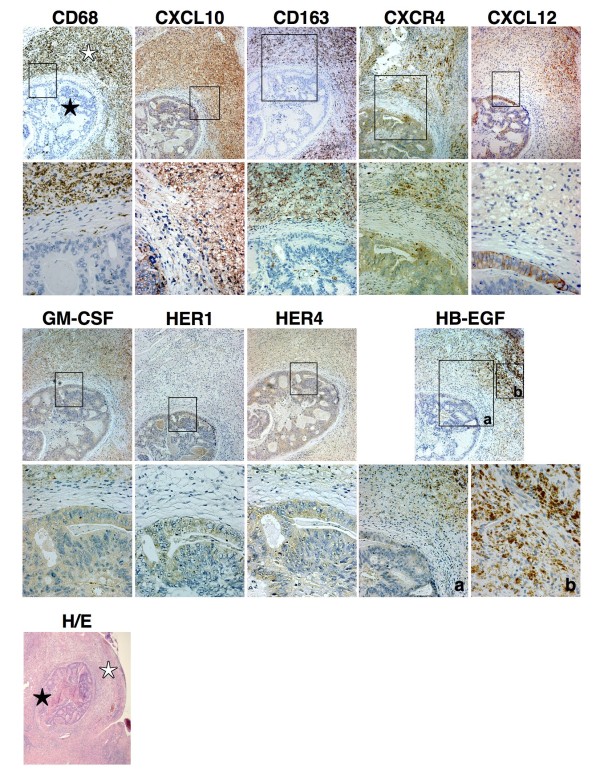
**Ligand/receptor repertoire in metastatic colon cancer and infiltrating macrophages**. Serial preparations of a surgically removed hepatic, subglissonian colon cancer nodule (★) were stained with Abs against the specified molecules. Infiltrating CD68-positive macrophages (☆), which bridge perivascular zones to gland-like structures built up by metastatic colon cancer cells, stained positive for CXCL10 (a M1-marker) and CD163 (a M2-marker) indicating a mixed M1/M2 environment. They preferentially stained positive for CXCR4, GM-CSF, HB-EGF and CXCL12. Cancer cells were positive for HER1, HER4, CXCR4 and CXCL12, and to a lesser extent, GM-CSF. The role of these molecules in the crosstalk between tumour-associated macrophages and cancer cells was evaluated in the following experiments. Boxes delineate regions shown below at higher magnification (400×). H/E: a haematoxylin/eosin staining of the metastatic nodule (★) showing its hepatic topography among macrophages (☆) at low magnification (40×). A representative case out of 15 is shown.

We analysed the histological pattern of surgical samples from 15 patients aged 60 to 79 who underwent hepatic lobectomy in order to excise metastatic colon cancer nodules. In Figure 1, we show the representative histology from a 76-year-old patient who had such a procedure. Serial preparations of a subglissonian metastatic nodule were stained by immunohistochemistry for CD68, CXCL10, CD163, CXCR4, CXCL12, GM-CSF, HER1, HER4 and HB-EGF. Macrophages, which were found to infiltrate the metastatic region often creating a bridge between perivascular zones and metastases, were intensely positive for CD68, CXCR4, GM-CSF and HB-EGF; some were CXCL12-positive. Of interest, macrophages stained positive for both CXCL10 (M1-marker) and CD163 (M2-marker). Though we could not perform a double staining, the distribution of the expression of CXCL10 and CD163 suggests a cellular co-expression rather than distinct populations of cells. In any case, the macrophages were not definitely polarized towards a typical M2 pattern but instead showed a mixed M1/M2 pattern. The cancer cells were positive for CXCR4, CXCL12, HER1, HER4 (a chemotactic receptor that responds to HB-EGF), and GM-CSF. The cellular distribution of ligands and receptors suggested specific interplays that were tested in the following experiments performed on the HeLa and DLD-1 cancer cell lines, which express the same pattern of molecules, and on human mononuclear phagocytes *ex vivo*.

### CXCL12 induces HB-EGF synthesis and release in mononuclear phagocytes

**Figure 2:**
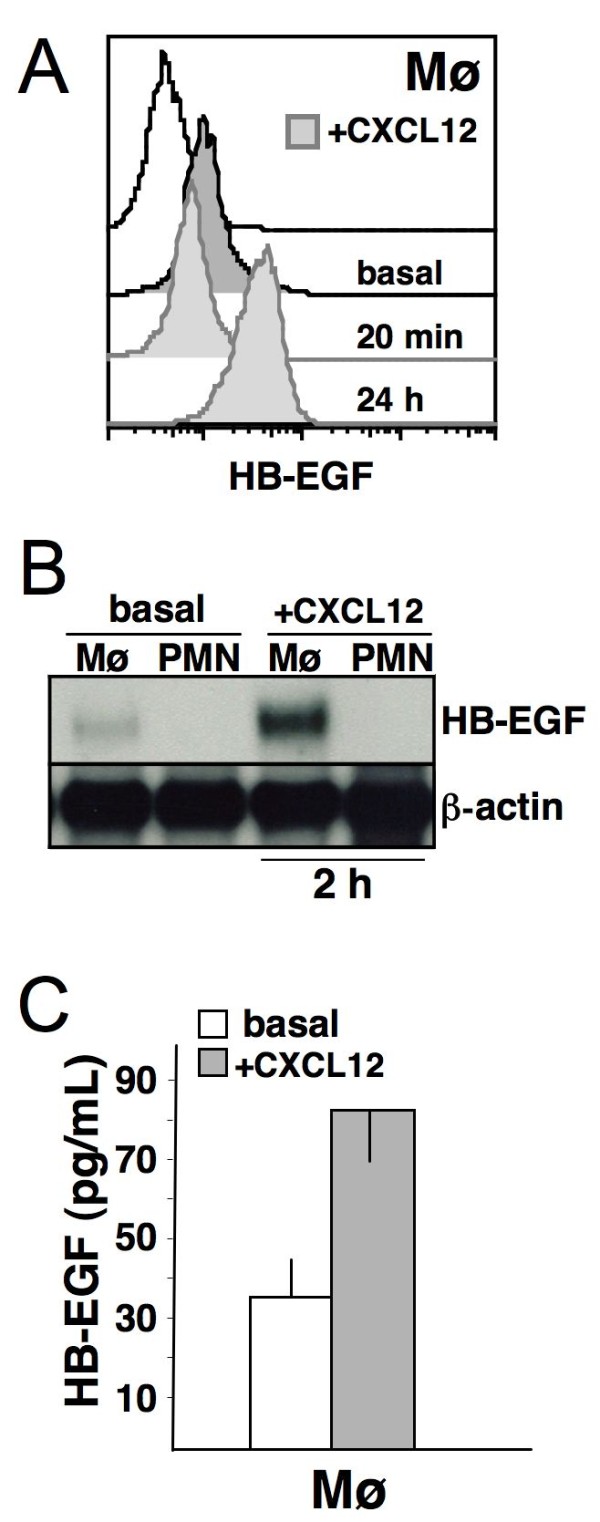
**CXCL12 modifies HB-EGF expression in mononuclear phagocytes**. Human mononuclear phagocytes (Mø) were cultured alone or in the presence of 200 ng/mL CXCL12. Cells were collected after 20 minutes, 2 hours and 24 hours; cell-free supernatants were collected after 24 hours and the levels of soluble HB-EGF protein were measured using a specific ELISA. (A) Flow cytometric analysis showing that CXCL12-stimulated Mø released HB-EGF (after 20 minutes) and up-regulated its expression (after 24 hours). (B) Northern blot analysis on Mø and neutrophils (PMN, used as negative control) collected after 2 hours of stimulation with CXCL12. Only Mø produced detectable levels of HB-EGF mRNA in basal conditions, and HB-EGF transcripts were up-regulated upon stimulation. After 24 hours, the mRNA up-regulation persisted (data not shown). (C) CXCL12 treatment induced Mø to release HB-EGF into the culture medium (*p *< 0.05). Representative pictures or the means ± SD out of 10 experiments are shown.

Under basal conditions, mononuclear phagocytes express HB-EGF (Figure 2A, B). When we stimulated mononuclear phagocytes with 200 ng/mL CXCL12, the membrane density of HB-EGF was initially reduced (at 20 minutes), but was increased after 24 hours (Figure 2A). After a 2-hour stimulation, constitutive HB-EGF mRNA transcripts were up-regulated in mononuclear phagocytes (Figure 2B), and this effect increased up to 24 hours (data not shown). This induction of HB-EGF was cell type specific. For instance, we show in Figure 2B that neutrophils did not express HB-EGF transcripts either basally or after CXCL12 stimulation. A 1-hour stimulation of mononuclear phagocytes with CXCL12 determined an increase in the HB-EGF concentration in the culture supernatants (Figure 2C). Thus, CXCL12-dependent signals induce mononuclear phagocytes to release HB-EGF from the cell membrane, increase the amounts of HB-EGF transcripts at 2 hours, and upregulate HB-EGF synthesis, leading to an increase in membrane-bound HB-EGF at 24 hours.

### HB-EGF-dependent HER1 phosphorylation

**Figure 3:**
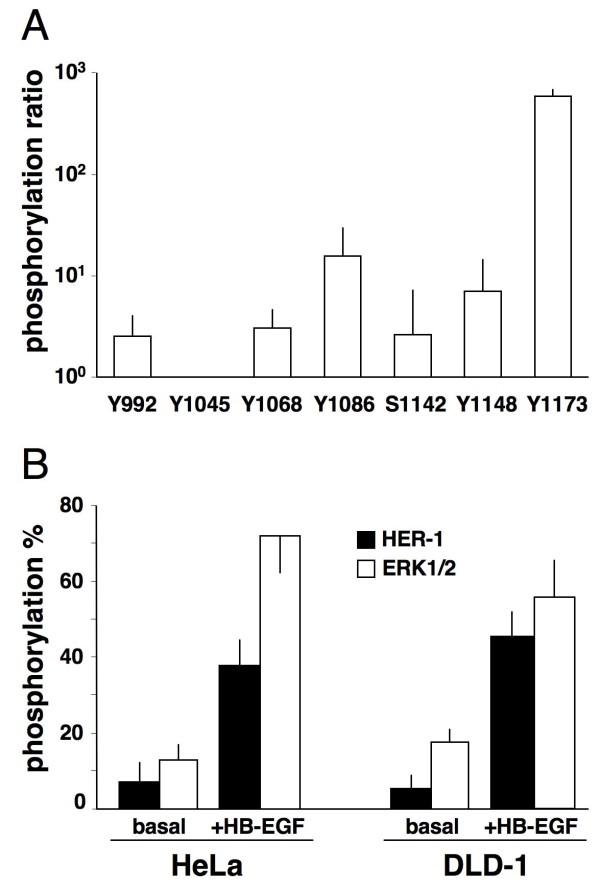
**HB-EGF-induced HER1 phosphorylation**. (A) HER1 autophosphorylation pattern derived from mass spectrometry analysis of trypsin-digested peptides from HeLa cells stimulated with 25 ng/mL HB-EGF for 20 minutes. Seven phosphorylation sites are represented as phosphorylation ratio (phosphorylation after stimulus/basal phosphorylation). (B) HeLa and DLD-1 cells were stimulated with 25 ng/mL HB-EGF for 20 minutes. Phosphorylation of HER1 and ERK1/2 was measured by ELISA and is expressed as phosphorylated molecules/total molecules and represented as per cent ratio. The means ± SD of 10 experiments are depicted.

To test if the stimulation of mononuclear phagocytes with CXCL12 could induce HER1 transactivation in bystander cells *via *HB-EGF shedding, we performed transwell co-cultures, in which we analysed HER1 phosphorylation at tyrosine 1068. Tyrosine 1068, a major site of autophosphorylation that is associated with the activation of Ras, MEK and ERK1/2 [24] was chosen after performing mass spectrometry analysis of ligand-dependent HER1 phosphorylation in HeLa cells. Mass spectrometry confirmed that 25 ng/mL HB-EGF induced a phosphorylation pattern different from that induced by other HER1 ligands [23,24] and that Y1068 phosphorylation was induced by HB-EGF in either HeLa or DLD-1 cells (Figure 3A). Moreover, HB-EGF-dependent phosphorylation was coupled to phosphorylation of ERK1/2 at threonine 185 and tyrosine 187 (Figure 3B), as expected [24].

### CXCL12-driven release of HB-EGF from mononuclear phagocytes transactivates HER1 and supports proliferative, anti-apoptotic and angiogenic effects in bystander cells

**Figure 4:**
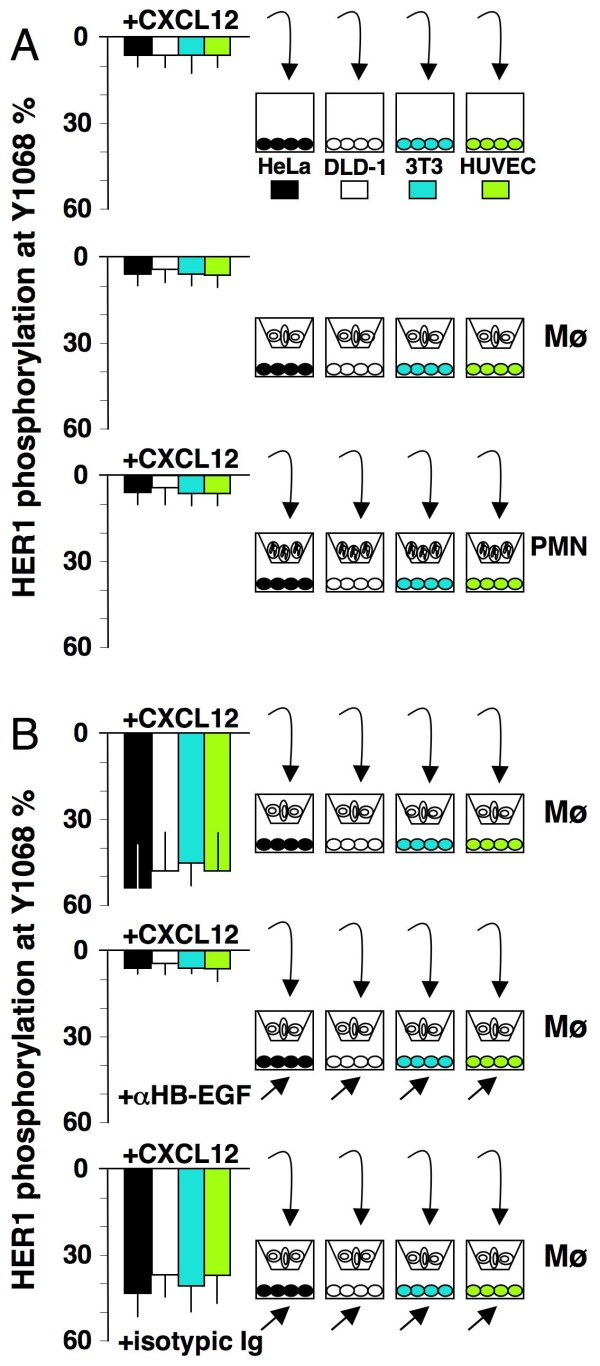
**CXCL12-dependent transactivation of HER1 in transwell experiments**. Human mononuclear phagocytes (Mø) and neutrophils (PMN, used as negative control) were stimulated with 200 ng/mL CXCL12 in the upper chamber of a transwell containing HeLa, DLD-1, Balb/c 3T3 or HUVEC target cells in the lower chamber. HeLa, DLD-1, Balb/c 3T3 and HUVEC were collected after 20 minutes of stimulation, and HER1 phosphorylation at Y1068 was measured by an ELISA using specific anti-tyrosine mAbs, expressed as phosphorylated molecules/total molecules and represented as a per cent ratio. (A) Negative controls: CXCL12 alone, stimulated PMN or unstimulated Mø were ineffectual. (B) Effect of Mø stimulation: CXCL12 led to HER1 transactivation in either HeLa, DLD-1, Balb/c 3T3 or HUVEC (*p *< 0.05). Pre-treatment with 100 μg/mL anti-HB-EGF neutralising Ab abolished Y1068 phosphorylation in the target cells (*p *< 0.05). The isotypic control of neutralising anti-HB-EGF mAb was not effective. The colour pattern of the bars refers to each type of target cell. The means ± SD of 10 experiments are depicted.

**Figure 5:**
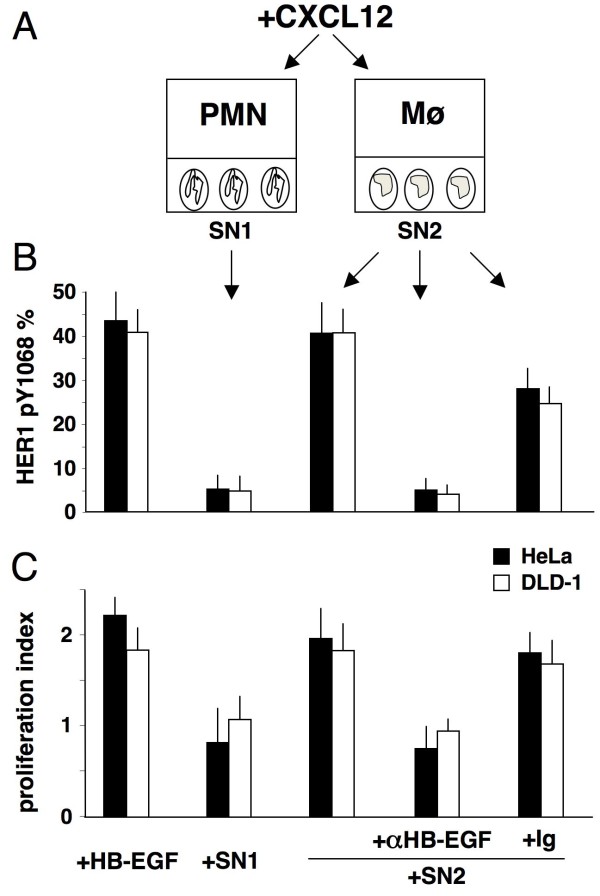
**Proliferation induced by supernatants from CXCL12-stimulated mononuclear phagocytes**. (A) Human mononuclear phagocytes (Mø) or neutrophils (PMN) were stimulated with 200 ng/mL CXCL12 and cell free supernatants were collected after 24 hours and added to either HeLa or DLD-1 cells. (B) Supernatants from CXCL12-stimulated PMN (SN1) were not effective at inducing phosphorylation of HER1, because PMN did not produce HB-EGF. Supernatants from CXCL12-stimulated Mø (SN2) induced HER1 phosphorylation at Y1068 when added to HeLa or DLD-1 cells (*p *< 0.05). The phosphorylation was genuinely inhibited by 100 μg/mL anti-HB-EGF neutralising Abs. (C) SN2 caused HeLa and DLD-1 cells to proliferate to a degree that was comparable to stimulation with 25 ng/mL HB-EGF. The means ± SD of 10 experiments are depicted.

**Figure 6:**
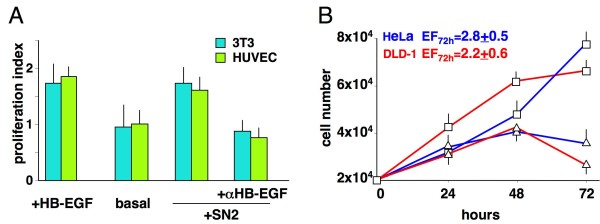
** Angiogenic, proliferative and anti-apoptotic activities of HB-EGF**. (A) HB-EGF or supernatants from CXCL12-stimulated mononuclear phagocytes (SN2) induced stromal (Balb/c 3T3) and endothelial (HUVEC) cells to proliferate, which shows that both HB-EGF and the evaluated supernatants share angiogenic potential. The supernatant-associated angiogenic signals were inhibited by 100 μg/mL anti-HB-EGF neutralising Abs (*p *< 0.05). (B) HB-EGF induced proliferation and anti-apoptotic effects (*p *< 0.05) in HeLa (*blue*) and DLD-1 (*red*) cells. Cultures were performed in serum free medium in the absence (△) or presence (□) of 25 ng/mL HB-EGF. Proliferation was evaluated by an MTT assay after 24, 48 and 72 hours in culture. Apoptosis was evaluated at 72 hours by the detection of internucleosomal DNA fragmentation by a specific ELISA. The ratio between absorbance of untreated and treated cells (enrichment factor, EF) was used as an index of rescue from apoptosis due to serum deprivation. The means ± SD of 5 experiments are depicted.

To determine if CXCL12 induces the transactivation of HER1, we performed the transwell experiments depicted in Figure 4. Mononuclear phagocytes (and neutrophils as negative control) in the upper chamber were stimulated with 200 ng/mL CXCL12 and HER1-positive HeLa, DLD-1, Balb/c 3T3 cells and HUVEC were used in the lower chamber as target cells. After 20 minutes, the cells were analysed to assess HER1 phosphorylation at tyrosine 1068. Figure 4A depicts several negative controls. The direct stimulation of HeLa, DLD-1, Balb/c 3T3 cells and HUVEC with CXCL12 did not induce HER1 phosphorylation. Unstimulated mononuclear phagocytes did not induce HER1 phosphorylation in the target cells. Neutrophils, which do not express HB-EGF [20], were treated with CXCL12 and this treatment did not lead to phosphorylation of HER1 at tyrosine 1068 in the target cells. In contrast, as depicted in Figure 4B, treatment of mononuclear phagocytes with CXCL12 led to the strong phosphorylation of Y1068 in all of the target cells. Pre-treatment with 100 μg/mL of neutralising anti-HB-EGF, but not its corresponding controls, inhibited the transactivation of HER1. Finally, supernatants from CXCL12-stimulated neutrophils, which did not produce HB-EGF, were not effective (SN1, Figure 5A, B, C). Mononuclear phagocytes-derived supernatants (SN2, Figure 5A) contained factors that led to HER1 phosphorylation in, and proliferation of, HeLa and DLD-1 cells (Figure 5B, C) as well as HUVEC and Balb/c 3T3 cell proliferation (SN2, Figure 6A). Blockade of HB-EGF with the neutralising Ab abolished the phosphorylation and the proliferation of the cells (Figures 4B; 5B, C; 6A). However, this effect did not occur when using indifferent isotypic immunoglobulins. Thus, CXCL12 induced the release of functional HB-EGF from mononuclear phagocytes, transactivation of HER1 and proliferation of cancer cells (HeLa and DLD-1), fibroblasts (Balb/c 3T3 cells) and endothelial cells (HUVEC). This occurred both in transwell co-cultures and after adding conditioned medium (from cultures of mononuclear phagocytes stimulated with CXCL12) to the target cells. Moreover, the metastatic colon cancer cells stained positive for HER4 (Figure 1), through which HB-EGF exerts powerful chemotactic activity [19]. Thus, HB-EGF can induce cancer cell chemotaxis and proliferation as well as microenvironment-targeted angiogenic signals. Finally, Figure 6B shows that HB-EGF conferred upon HeLa and DLD-1 cells both proliferative and anti-apoptotic signals; these latter signals clearly emerged under starvation conditions, as indicated by the statistically significant reduction in mono/oligonucleosomes released into the cytoplasm.

### CXCL12 and HB-EGF induce cancer cells to synthetise and release GM-CSF

**Figure 7:**
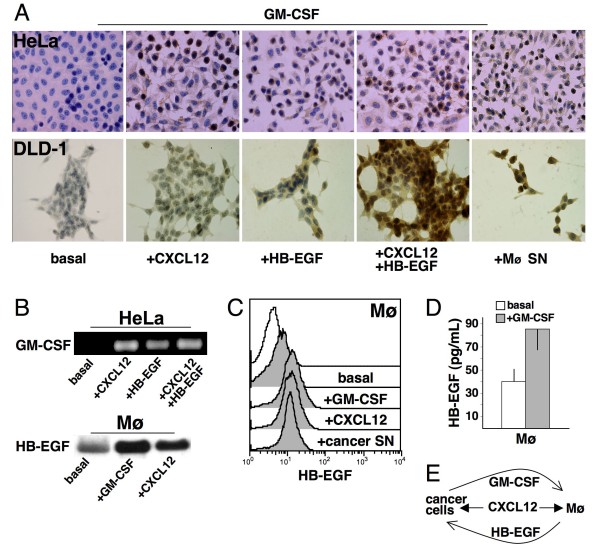
**GM-CSF/HB-EGF paracrine loop between mononuclear phagocytes and cancer cells**. (A) HeLa and DLD-1 cells produced GM-CSF following stimulation with 200 ng/mL CXCL12 and/or 25 ng/mL HB-EGF or supernatants obtained by treating human mononuclear phagocytes (Mø SN) with 25 ng/mL GM-CSF for 24 hours. (B) RT-PCR for GM-CSF in HeLa cells and Northern blot for HB-EGF in Mø stimulated as noted in the captions. (C) Mø upregulated HB-EGF surface expression following stimulation with GM-CSF, CXCL12 or cancer supernatants (cancer SN) that were obtained by GM-CSF-positive HeLa/DLD-1 cells. (D) Mø released HB-EGF (*p *< 0.05) upon GM-CSF or CXCL12 stimulation, as measured by a specific ELISA. Representative pictures or the means ± SD out of 10 experiments are shown. (E) Mechanistic representation of paracrine loops as suggested by the experimental findings.

**Figure 8:**
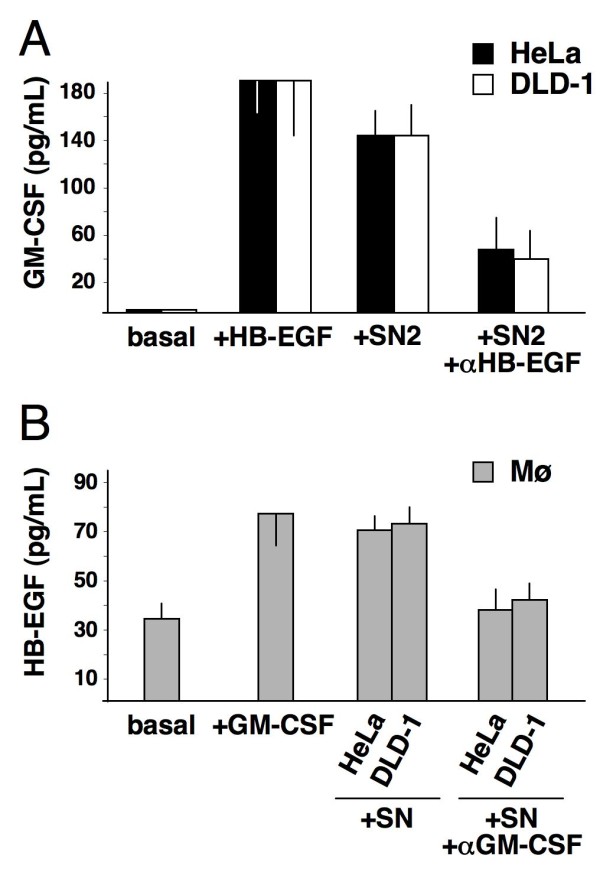
**GM-CSF and HB-EGF release from cancer cells and mononuclear phagocytes**. ELISA analysis (see Methods section) revealed that GM-CSF and HB-EGF were barely detectable under basal conditions in supernatants from HeLa/DLD-1 cells or mononuclear phagocytes, respectively, but accumulated 24 hours after stimulation. (A) Stimulation of HeLa/DLD-1 with 25 ng/mL HB-EGF or supernatant from mononuclear phagocytes (SN2) induced GM-CSF release into supernatants. 100 μg/mL anti-HB-EGF blocking Abs reduced the supernatant-dependent release (*p *< 0.05). (B) Stimulation of mononuclear phagocytes with 25 ng/mL GM-CSF or supernatant from GM-CSF-positive cancer cells (SN) led to the accumulation of HB-EGF in supernatants. 100 μg/mL anti-GM-CSF neutralising Abs suppressed the supernatant-dependent release (*p *< 0.05). The means ± SD out of 10 experiments are depicted.

In addition, when HeLa and DLD-1 cancer cells were stimulated with 200 ng/mL CXCL12 and/or 25 ng/mL HB-EGF, GM-CSF proteins were detected by immunocytochemistry after 24 hours and new GM-CSF transcripts (as assessed by RT-PCR) appeared after 2 hours (Figure 7A, B). Conditioned medium obtained from cancer cells contained GM-CSF (Figure 8A) and induced HB-EGF expression in, and release from, mononuclear phagocytes (Figures 7C; 8B). Inhibitory anti-GM-CSF mAbs significantly reduced the production of HB-EGF (Figure 8B). Thus, CXCL12 and HB-EGF induced GM-CSF expression in HeLa and DLD-1 cancer cells.

### Paracrine loop activated by CXCL12

As described above, CXCL12 was shown to prompt mononuclear phagocytes and cancer cells to release HB-EGF and GM-CSF, respectively. On the other hand, we have previous evidence showing that GM-CSF is a strong inducer of HB-EGF expression in mononuclear phagocytes [19,20]. If HB-EGF released by mononuclear phagocytes can trigger the production of GM-CSF in cancer cells, a possible GM-CSF/HB-EGF paracrine loop may exist that is initially activated by CXCL12. Thus, we tested (i) HeLa and DLD-1 cancer cells for the production of GM-CSF upon HB-EGF stimulation and (ii) mononuclear phagocytes for the production of HB-EGF upon GM-CSF stimulation. This choice was based on the known differential receptor expression in mononuclear phagocytes, as opposed to cancer cells, which are usually negative for the GM-CSF receptor. Figure 7 depicts the experiments suggesting that a paracrine loop exists between Mø and HeLa or DLD-1 cancer cells. When these cancer cells were stimulated with 200 ng/mL CXCL12 and/or 25 ng/mL HB-EGF, they produced and released GM-CSF (Figures 7A, B; 8A). When mononuclear phagocytes were stimulated with CXCL12 and/or 25 ng/mL GM-CSF, they produced and released HB-EGF (Figures 2; 7B, C, D; 8B). HB-EGF mRNA transcripts and membrane protein levels were increased after 2 hours (Figures 2B; 7B) and after 24 hours of stimulation (Figures 2A, C; 7C, D; 8B). These results were reproduced by the addition of conditioned medium from mononuclear phagocytes to cancer cells and *vice versa *(Figures 7A, C; 8). Inhibitory anti-HB-EGF and anti-GM-CSF Abs significantly reduced the production of GM-CSF by cancer cells (Figure 8A) and HB-EGF by mononuclear phagocytes (Figure 8B), respectively. Thus, a paracrine loop may exist between mononuclear phagocytes and HeLa or DLD-1 cancer cells, as exemplified in Figure 7E. CXCL12 seems to act as an additive or synergistic agent in this positive feedback loop. The expression of these molecules as found in patient biopsy samples (Figure 1) is in line with and corroborates our findings *in vitro*.

### Blockade of GM-CSF in cancer cells

**Figure 9:**
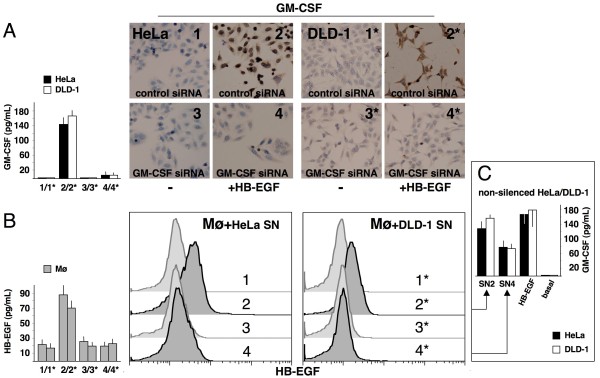
**Knockdown of GM-CSF protein levels after siRNA application in cancer cells**. HeLa/DLD-1 cells were transfected with control siRNA (1/1*, 2/2*) or GM-CSF siRNA (3/3*, 4/4*) and cultured in the absence or presence of 25 ng/mL HB-EGF. The numbers indicate the culture conditions and the corresponding supernatants (SN) used for ELISA or cell stimulation. (A) Blockade of GM-CSF production in cultures of HeLa/DLD-1 cells transfected with GM-CSF siRNA was confirmed by immunocytochemistry (2/2* vs. 4/4*) and ELISA (*left side*; 2/2* vs. 4/4*, *p *< 0.05). (B) SN from GM-CSF-silenced HeLa/DLD-1 did not induce HB-EGF expression in mononuclear phagocytes (Mø), as revealed by flow cytometry (2/2* vs. 4/4*) and ELISA (*left side*; 2/2* vs. 4/4*, *p *< 0.05). (C) Mø stimulated with SN from GM-CSF-silenced HeLa/DLD-1 cells released SN less effective at inducing GM-CSF in non-silenced cancer cells, as determined by ELISA (see Methods section; SN2 vs. SN4, *p *< 0.05). Representative pictures or the means ± SD out of 5 experiments are shown.

To further test the GM-CSF/HB-EGF loop between cancer cells and macrophages, we established a siRNA sequence targeted against GM-CSF. This siRNA efficiently blocked GM-CSF induction and release in HeLa and DLD-1 cells in response to both HB-EGF (Figure 9A) and conditioned medium from mononuclear phagocytes (data not shown). Similarly, inhibitory anti-HB-EGF Abs significantly reduced the production of GM-CSF by cancer cells (Figure 8A). Therefore, HB-EGF genuinely and specifically induced GM-CSF expression in these cells. The supernatant from silenced cancer cells lost the ability to upregulate HB-EGF in mononuclear phagocytes (Figure 9B) and inhibitory anti-GM-CSF Abs blocked the induction of HB-EGF (Figure 8B), showing that the upregulation was specifically due to GM-CSF. Finally, mononuclear phagocytes treated with supernatants from GM-CSF-silenced cancer cells lost the ability to induce GM-CSF in non-silenced cancer cells (Figure 9C). Thus, GM-CSF silencing data complement the ligand-blocking experiments obtained with inhibitory Abs, and strengthen our experimental evidence supporting the existence of an activated GM-CSF/HB-EGF loop between cancer cells and mononuclear phagocytes. When available, HB-EGF specifically stimulates cancer cells to produce GM-CSF, and the reciprocal availability of the two factors activates a positive feedback loop between them (Figure 7E).

## Discussion

The current study defines a novel mechanism whereby CXCL12 redirects macrophages to promote a microenvironment that is suitable for cancer survival *via *a GM-CSF/HB-EGF paracrine loop. To our knowledge, there are no other studies showing that human mononuclear phagocytes release and up-regulate HB-EGF, while cancer cells release and upregulate GM-CSF, when stimulated with CXCL12.

By evaluating histological samples from human colon cancer metastases in the liver, we observed that numerous HB-EGF/CXCR4-positive macrophages, which expressed both the M1 CXCL10 and the M2 CD163 markers, indicating a mixed M1/M2 microenvironment, infiltrated metastatic cancer cells. These in turn were positive for CXCR4, CXCL12, GM-CSF and HER1 (Figure 1). We then validated the mutual interactions associated with this repertoire of molecules in standard and transwell experiments performed on human mononuclear phagocytes and HeLa and DLD-1 cancer cell lines, expressing the same molecules in the same cellular distribution as macrophages and cancer in biopsy samples.

CXCL12 and GM-CSF induced mononuclear phagocytes to synthetise and release HB-EGF. Northern blotting of RNA from kinetic experiments revealed that maximal expression of HB-EGF mRNA occurred between 2 and 24 hours after CXCL12- or GM-CSF-dependent induction, leading to an increase in membrane HB-EGF molecule density (Figures 2; 7B, C). In transwell experiments, CXCL12-stimulated mononuclear phagocytes released HB-EGF that caused the phosphorylation of HER1 in HeLa and DLD-1 target cells (Figure 4B). Cell-free supernatants from CXCL12-treated mononuclear phagocytes induced HER1 phosphorylation followed by cellular proliferation in either HeLa or DLD-1 cells, an effect that was inhibited by anti-HB-EGF neutralising Abs (Figure 5).

Stimulation with CXCL12, HB-EGF or both induced GM-CSF transcripts in HeLa and DLD-1 cells. At 24 hours, immunocytochemistry revealed clear-cut staining for GM-CSF in both cell lines (Figure 7A). Their conditioned medium contained GM-CSF that induced Mø to produce HB-EGF (Figures 7C; 8B). Conversely, mononuclear phagocytes conditioned medium contained HB-EGF that induced cancer cells to produce GM-CSF (Figures 7A; 8A). These effects were largely counteracted by the addition of specific neutralising Abs (Figure 8) or by GM-CSF silencing (Figure 9). In conclusion, CXCL12 induced HB-EGF in mononuclear phagocytes and GM-CSF in HeLa and DLD-1 cancer cells, activating or enhancing a GM-CSF/HB-EGF paracrine loop.

Thus, we have evidence for a specific pathway of activation in mononuclear phagocytes (CXCL12-stimulated Mø release of HB-EGF) that may match the specific biological properties of some cancers (HeLa, DLD-1 and metastatic colon cancer). We have also documented a specific pathway of activation in cancer cells (CXCL12/HB-EGF-stimulated cancer cell release of GM-CSF) that may match the specific biological properties of mononuclear phagocytes. This interplay between mononuclear phagocytes and cancer cells may lead to an inflammatory environment that favours rather than inhibits tumour growth. Furthermore, both macrophages and cancer cells were activated upon CXCL12 stimulation in liver biopsies (Figure 1), though we could not conclusively establish whether cancer cells produced their own CXCL12 or merely internalized CXCL12, produced by stromal cells. Other studies have demonstrated that CXCL12 transactivates HER2 in breast cancer cells [25], enhancing the expression of CXCR4 and favouring metastases [11]. In our work, CXCL12 has been shown to transactivate HER1 and induce GM-CSF. The latter is a specific inducer of HB-EGF, which in turn binds to HER1. HB-EGF acts as a chemotactic, pro-growth and anti-apoptotic factor in cancer cells, and plays a role as angiogenic factor by inducing endothelial cells and fibroblasts to proliferate (Figure 6). It also promotes angiogenesis by induction of VEGF [20]. In general, HB-EGF is a powerful inducer of fibroblast activities [17,19,20] that are involved in orchestrating inflammation and promoting tumour growth, angiogenesis and recruitment of macrophages and cancer cells [7].

Therefore, the CXCL12 receptors, CXCR4 and CXCR7, should be thought of as a node that connects multiple loops [26-30], including the highly important EGF/HER loops [13], linking cancer (oncogenes) and inflammation [5]. Based on our previous demonstration of the role of HER1 in the regulation of mesenchymal stem cell proliferation and differentiation [16], as well as on some general models [5,31,32], we speculate that the crosstalk between CXCL12/CXCR4 and HB-EGF/HERs may contribute to the balance between the HER1-dependent cellular responses of differentiation and self-renewal [31-34].

## Hypothesis

This study provides evidence that CXCL12 partecipates in the selective production of cytokines, leading to a GM-CSF/HB-EGF paracrine loop that may favour neoplastic growth. CXCL12 has chemotactic activity towards cancer and immune cells; in both cell types, it induces cytokines that retain pro-tumour activity and modulate the stromal component [7,16], contributing to a tumour-permissive microenvironment. Therefore, CXCL12 signalling may provide a unifying basis for better understanding the complex relationships between cancer and inflammatory cells in terms of receptor crosstalk. For instance, the involvement of mixed M1/M2, GM-CSF-stimulated macrophages in a tumour-promoting loop challenges the view of tumour-permissive macrophages as polarized M2 mononuclear phagocytes and hints at contexts in which pro-inflammatory microenvironments may allow effective tumour-promoting activities through mediators such as GM-CSF and HB-EGF. These mediators, indeed, seem to play a role that favours both cancer and a shift towards a M1-polarized microenvironment. Eventually, such an understanding could contribute to the development of tools to interfere with recognised pathogenic signals [35].

## Competing interests

The authors declare that they have no competing interests.

## Authors' contributions

AR and MG performed the research, analysed the data, and performed statistical analyses; AZ, ED, PM and MB performed the research and contributed vital reagents and analytical tools; MK, GP contributed criticism; FV designed the research, analysed and interpreted data, and wrote the paper; all authors checked the final version of the manuscript.
